# Evaluation of Secondary Metabolites in Various Stages of Okra Seed Development and Their Anti-Inflammatory Potential Against IL-1ß

**DOI:** 10.3390/ijms27157002

**Published:** 2026-08-04

**Authors:** Lorrenne Caburatan, Joonho Park

**Affiliations:** 1 Department of Biological Sciences, Mindanao State University-Iligan Institute of Technology, Iligan City 9200, Philippines; lorrenne.caburatan@g.msuiit.edu.ph; 2 Department of Fine Chemistry, Seoul National University of Science and Technology, 232-Gongneung-ro, Nowon-gu, Seoul 01811, Republic of Korea; 3 Center for Functional Biomaterials, Seoul National University of Science and Technology, 232-Gongneung-ro, Nowon-gu, Seoul 01811, Republic of Korea

**Keywords:** Okra seeds, secondary metabolites, NO production, pro-inflammatory cytokine, IL-1β

## Abstract

*Okra* is a flavonoid-rich food known to confer a variety of health benefits and have extensive therapeutic characteristics. In this study, okra seeds at five stages of development (youngest, young, intermediate, mature and fully mature) were assessed for secondary metabolite content using 70% methanol. Extracts from mature seeds were found to contain the highest accumulation of total polyphenol content (TPC) (536.65 ± 80.13 mg GAE/g), total flavonoid content (TFC) (234.73 ± 45.58 mg ISE/g), DPPH scavenging activity (70.11%) and ABTS+ activity (75.19%). High-performance liquid chromatography (HPLC) analysis revealed that in fully mature seed extracts, quercetin (148.14 ± 11.01 mg/kg) is abundant, while isoquercetin and quercertin-3-O-gentibiose are respectively abundant in the youngest (5205.02 ± 48.54 mg/kg) and mature seeds (12,396.48 ± 77.54 mg/kg). Mature seed extracts also exhibited the highest activity against nitric oxide (NO) production and the pro-inflammatory cytokine interleukin-1 beta (IL-1β) in lipopolysaccharide-induced (LPS) RAW 264.7 cells. The findings from this study showed that secondary metabolite accumulation in okra seeds varies at different stages of development. Thus, determining the bioactive compounds at different levels of seed development is considered essential for potential pharmacological advancements in biologically significant plants.

## 1. Introduction

Okra (*Abelmoschus esculentus*) is an edible plant known to contain a number of secondary metabolites that have several pharmacological effects. It is native to Africa and is mostly used for culinary purposes in tropical and subtropical regions such as the Mediterranean, India, and Southeast Asia [1,2]. Previous studies have been conducted on the pharmacological effects of okra pods, flowers, and seeds. Okra pods have been known to have beneficial effects against ailments such as gastric ulcer, hepatitis, and colitis [3]. Okra flowers also contain a high content of total flavonoids and have been investigated for their antitumor potential against colorectal cancer [4]. Okra seeds are also known for their potent phenols, which exhibit radical scavenging activity and anti-fatigue and anti-cancer effects, among others [1,2,5].

Stages in okra seed development can be classified primarily by the day of flowering, or simply the day the fruit emerges, as in the case of [6]. Seeds of okra are known to contain mainly quercetin derivatives, catechins and some endogenous gibberellins [7,8]. Their beneficial effects are due to the presence of secondary metabolites in the different tissues of okra.

Secondary metabolites are small bioactive molecules that play a role in the interaction of plants with their environment. These biomolecules are produced as a mechanism for plants to protect themselves against stressors and other related environmental stimuli [9]. Plants produce different types of secondary metabolites and their varied concentrations depend on the species, genotype, physiology, environmental factors at play during growth, and stage of development [10]. Studies on the anatomical differences in okra seeds at each stage of development are not yet available, and hence, studies on seed development in such cases are limited to the day after flowering.

Quercetin (3,3′,4′,5,7-pentahydroxyflavone) is a flavonoid known to be highly bioavailable [11] and is a compound found in okra seeds [12]. It acts as a natural chemo-preventive agent [13] and has been known to play roles in cell apoptosis and autophagy, senescence, deregulation of cellular energetics, inhibition of invasive metastasis and tumor-promoting inflammation, as well as antiangiogenic and cancer cell antiproliferative and growth suppressive effects, and hypertensive effects [11,14,15,16]. Consuming food that may contain these bioactive compounds, including catechins and anthocyanins, may also reduce risk of cardiovascular disease, diabetes, obesity, and hyperlipidemia, among others [12,17,18,19].

Moreover, quercetin has a number of molecular derivatives, including isoquercetin. Isoquercetin, or quercetin-3-O-glucoside, is a flavonol glucoside which is also a major flavonoid found in okra seeds. Isoquercetin has been reported to have antidiabetic effects in in vitro studies through increasing glucose uptake [12,13,14,15,16,17,18,19,20]. It has been known to inhibit α-glucosidase [21,22,23]. In a study of rat intestines, isoquercetin was found to selectively inhibit intestinal maltase and sucrose 6–10 times more than its related diglucoside [24]. Previous studies claimed that it exhibited anti-inflammatory and anti-apoptotic activity, regulation of glucose metabolism, antidiabetic, and antioxidant activity [12,13,14,15,16,17,18,19,20].

Quercetin-3-O-gentiobiose is a flavonol glycoside (5,7,30,40-tetrahydroxy-3-O-[b-D-glucopyranosyl-(1→6)]-β-D-glucopyranosideflavonol) [25]. It is another quercetin derivative found in okra seeds, which also possesses antioxidative and anti-fatigue properties [2,25]. It was shown to exert vasoprotective and anti-fatigue effects in a study on swimming mice by alleviating impairments of the vascular tissues; lessening the levels of inflammatory cytokines such as monocyte chemoattractant protein-1 (MCP-1), interleukin-6 (IL-6), and tumor necrosis factor-alpha (TNF-α); enhancing antioxidant enzyme activities; and increasing glucose tolerance in obese mice by reducing levels of blood glucose and serum insulin [25,26].

Determining whether amounts of quercetin and its derivatives are significant in varied stages of development in okra seeds will provide vital information that may elucidate further benefits that can be gained from levels of development in okra seeds.

The objective of this study is to therefore optimize the accumulation of secondary metabolites in okra using seeds in different stages of development and 70% methanol extractions while evaluating its effects in LPS-induced (lipopolysaccharide-induced) RAW 264.7 cell lines. Optimizing secondary metabolite production is deemed essential for possible pharmacological advances in biologically significant plants.

## 2. Results

### 2.1. Comparison of Solvent Extraction in Okra Seeds Using Methanol in Different Concentrations

Mature samples of okra seeds were extracted with different dilutions of methanol. Extraction of plant extracts using various solvents is a vital step in the isolation and recovery of bioactive materials prior to any analysis that is to be considered. A number of factors influence the accuracy and effectiveness of extraction, including the method employed, chemical nature of solvents, particle size of samples, possible presence of interfering substances, temperature, pH, volume ratio of sample to solvent, and the intervals of each extraction step [27].

**Figure 1:**
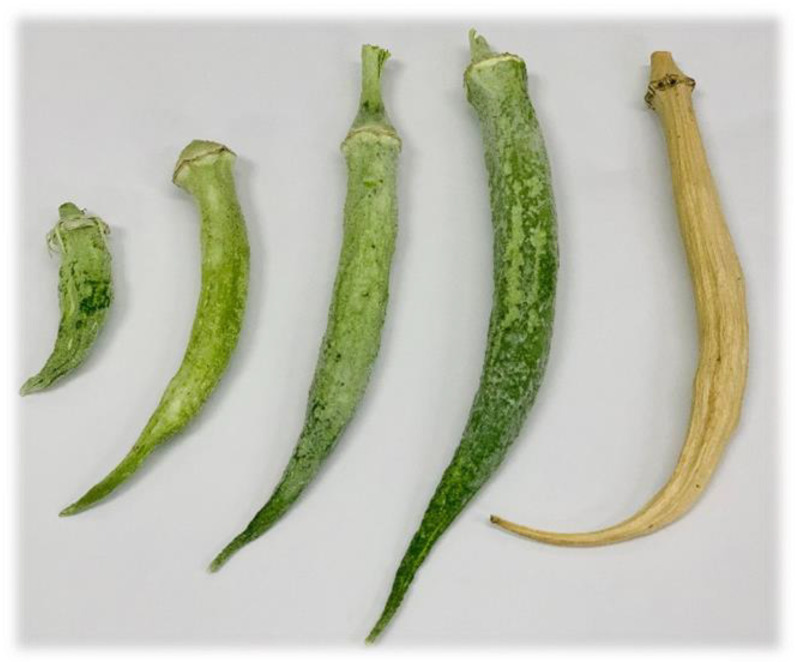
Okra fruit stages of development from when the fruit started to emerge. From left to right: youngest (YT), 5 days old; young (Y), 7 days old; intermediate (I), 9 days old; mature (M), 11 days old; and fully mature (FM), 20 days old.

Only mature okra seeds in this study were tested for the best possible extraction using various concentrations of methanol. Figure 1 shows okra fruits at different developmental stages, from the youngest (YT) to fully mature (FM). Results showed that the highest total polyphenol and flavonoid contents were obtained from 70% aqueous methanol extract (678.34 ± 6.15 mg GAE/g and 152.70 ± 1.24 mg ISE/g, respectively). Hence, 70% methanol extraction was used throughout the experiments. The extraction efficiency of each methanol solvent concentration is shown in Table 1.

### 2.2. Secondary Metabolite Content and Antioxidant Activity of Okra Seeds

**Figure 2:**
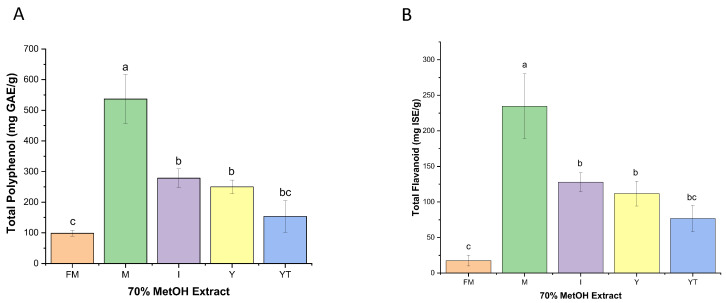
Total polyphenol content in mg GAE/g (**A**) and total flavonoids content in mg ISE/g (**B**) in okra seed extracts at various stages of development. Samples were derived from 70% methanol extracts of fully mature (FM), mature (M), intermediate (I), young (Y), and youngest (YT) seeds. Bars with different letters showed significant differences at *p* < 0.05.

The total polyphenol and flavonoid contents were found to be highest in the 70% methanol extract of mature okra seeds (536.65 ± 80.13 mg GAE/g and 234.73 ± 45.58 mg ISE/g, respectively), while the lowest contents were found in fully mature okra seeds (98.62 ± 9.63 mg GAE/g and 17.64 ± 7.54 mg ISE/g, respectively). Total polyphenol contents of mature seed extracts were around 48.14%~81.62%, which is significantly higher than the other samples; the total flavonoids content was around 45.51%~92.49% (Figure 2).

**Figure 3:**
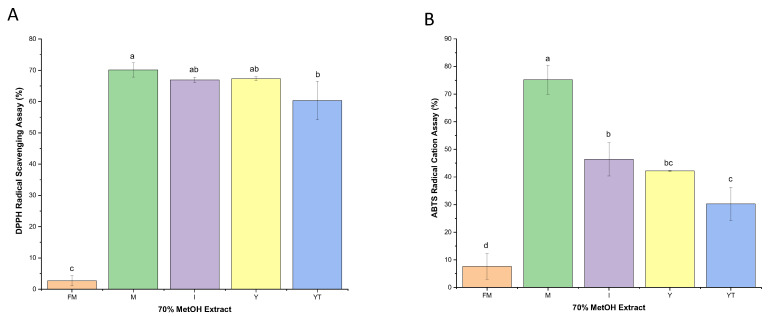
Percent of DPPH radical scavenging activity (**A**) and ABTS radical cation scavenging activity (**B**) in okra in various stages of seed development (FM, M, I, Y, and YT) for 70% methanol extraction. Bars with different letters showed significant differences at *p* < 0.05.

Antioxidant activity of okra seed extracts was evaluated for both DPPH radical scavenging activity and ABTS+ radical activity. Samples from mature seeds with 70% methanol extract were found to contain the highest activity for both antioxidant activity measures. Increases range from 4.02% to 95.98% for DPPH scavenging activity and 38.32% to 89.85% for ABTS+ radical activity (Figure 3). Hence, it can be noted that mature seed extracts from 70% methanol have the highest secondary metabolite content (TPC and TFC) as well as the highest percentage in antioxidant activity among other seed stages of development. The TPC and TFC are in a quantitative sense directly proportional to the antioxidant activity of mature okra seeds; it is already known that polyphenols and flavonoids have antioxidant properties to some extent.

### 2.3. Quantification of Quercetin, Isoquercetin, and Quercetin-3-O-Gentiobiose Content in Okra Seeds

Quercetin and isoquercetin were quantified from peak chromatogram areas extracted at 360 nm with retention times of around 17.4 and 22.4, respectively. The correlation coefficient (r^2^) for quercetin was 0.999 and 1 for isoquercetin, indicating good linearity. Figure S1 shows the representative HPLC chromatograms of okra seed extracts in 70% methanol.

**Figure 4:**
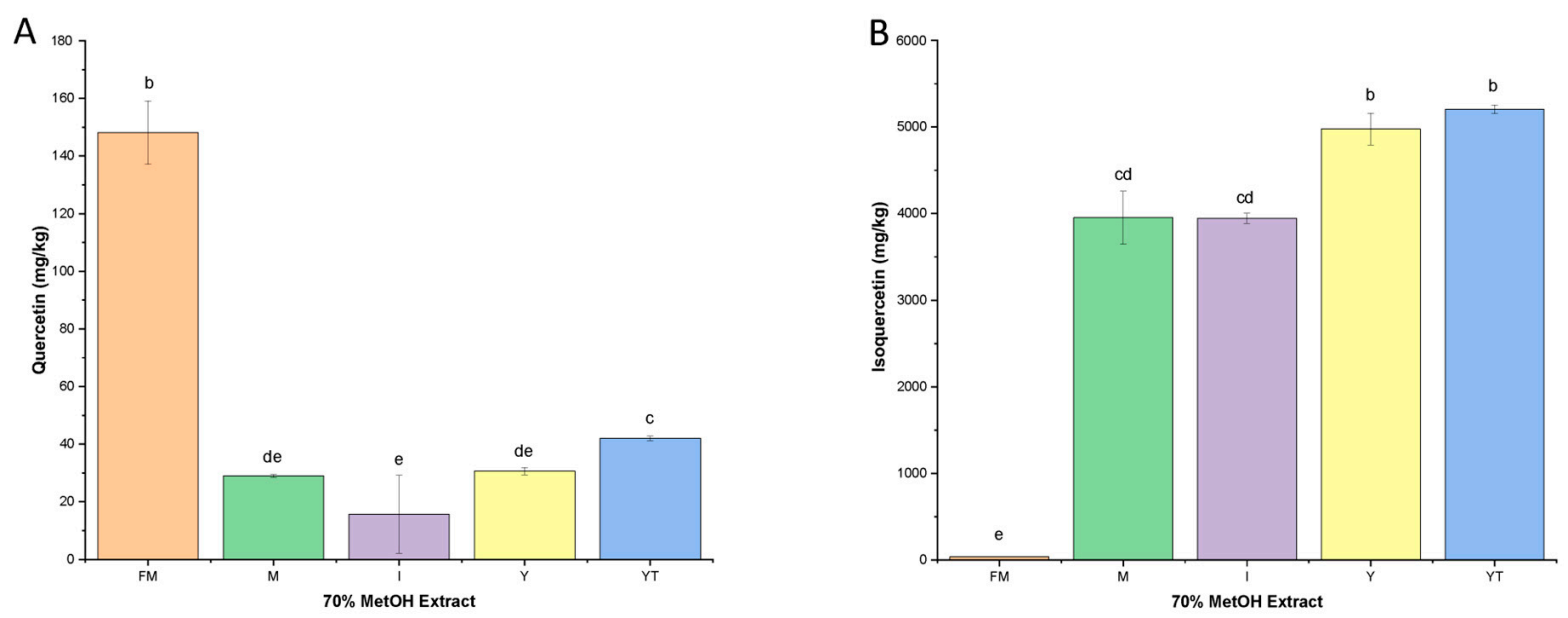
(**A**) Quercetin and (**B**) isoquercetin contents of okra seeds in various stages of development (FM, M, I, Y, and YT) for 70% methanol extraction. Significant differences at *p* < 0.05 are represented by bars with different letters.

Quercetin content varied significantly across developmental stages (Welch’s ANOVA F = 156.23, df = 4, 4.1, *p* < 0.001). Fully mature seeds contained the highest quercetin levels (148.14 ± 6.20 mg/kg), which were significantly higher than all other stages (*p* < 0.001 for all comparisons) (Figure 4A). The youngest seeds (42.05 ± 0.77 mg/kg) contained significantly more quercetin than mature (29.02 ± 0.46 mg/kg, *p* < 0.001), intermediate (22.86 ± 0.65 mg/kg, *p* < 0.001), and young seeds (30.63 ± 1.31 mg/kg, *p* < 0.001). No significant difference was observed between mature and young seeds (*p* = 0.356). These differences in the content of certain compounds at a particular stage of development are normal, as developmental factors are influential in the initiation and subsequent differentiation of cellular structures; they are particularly involved in the biosynthesis of secondary metabolites as well as their storage [28,29,30]. Normality was also assessed using the Shapiro–Wilk test, which confirmed normal distribution for all groups (*p* > 0.05). However, Levene’s test indicated unequal variances across stages (*p* = 0.001) (Table 2). Therefore, Welch’s ANOVA was used instead of the standard one-way ANOVA. Post hoc comparisons were performed using the Games–Howell test, which does not assume equal variances. Statistical significance was set at *p* < 0.05.

On the other hand, isoquercetin content varied significantly across developmental stages (Kruskal–Wallis H = 12.54, df = 4, *p* = 0.014). The highest isoquercetin levels were found in the youngest seeds (5205.02 ± 48.54 mg/kg), followed by young (4976.11 ± 181.85 mg/kg), intermediate (3956.93 ± 59.36 mg/kg), mature (3953.37 ± 308.26 mg/kg), and fully mature seeds (34.64 ± 2.29 mg/kg) (Figure 4B). Normality was also assessed using the Shapiro–Wilk test. The Young stage showed a non-normal distribution (*p* = 0.005), and Levene’s test indicated unequal variances across stages (*p* = 0.003). Therefore, the non-parametric Kruskal–Wallis test was used instead of one-way ANOVA (Table 3).

Quercetin-3-O-gentiobiose was quantified from the peak chromatogram area at 354 nm with a retention time of around 18.57. The correlation coefficient (r^2^) for quercetin-3-O-gentiobiose was 0.997, indicating good linearity. Figure S2 shows the representative HPLC chromatograms of okra seed extracts in 70% methanol.

**Figure 5:**
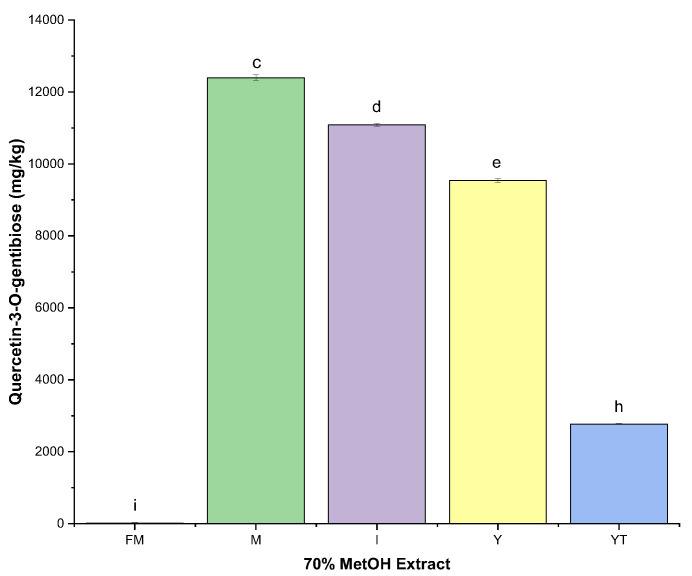
Quercetin-3-O-gentiobiose content of okra seeds in various stages of development (FM, M, I, Y, and YT) in 70% methanol extraction. Significant differences at *p* < 0.05 are represented by bars with different letters.

Quercetin-3-O-gentiobiose content was highest in mature seeds (12,396.48 ± 77.54 mg/kg), followed by intermediate (11,086.93 ± 49.54 mg/kg), young (9538.85 ± 57.47 mg/kg), youngest (2768.06 ± 10.31 mg/kg), and fully mature seeds (15.30 ± 14.80 mg/kg) (Figure 5). Both normality (Shapiro–Wilk *p* > 0.05) and equal variance (Levene’s *p* = 0.083) assumptions were met, allowing for one-way ANOVA, which revealed highly significant differences across stages (F = 98.45, df = 4, 10, *p* < 0.001). Tukey’s HSD post hoc test confirmed that all pairwise comparisons were statistically significant (*p* < 0.001 for all comparisons), indicating a progressive and distinct accumulation profile for each developmental stage (Table 4).

### 2.4. Effect of Okra Seed Extracts on RAW 264.7 Cells

**Figure 6:**
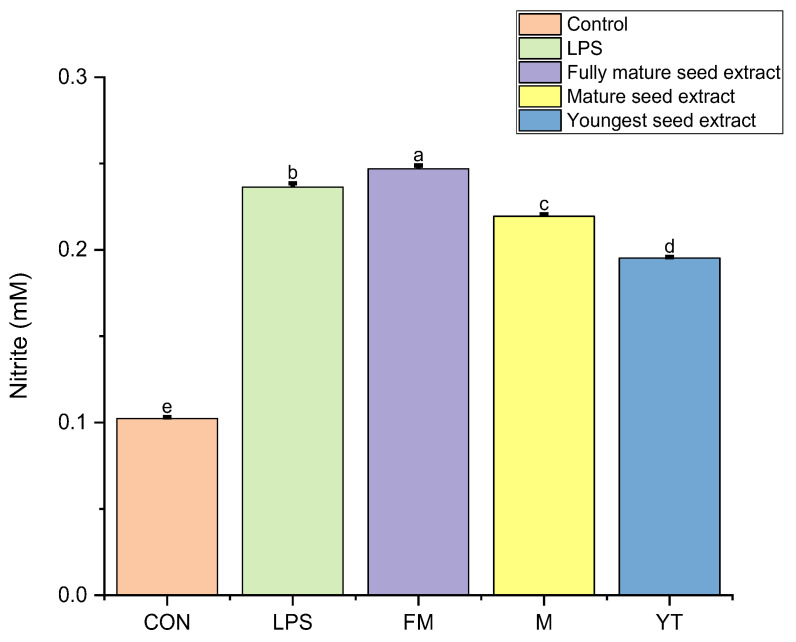
Nitric oxide levels in LPS-induced RAW 264.7 cells subsequently added to okra seed extracts in 70% methanol from different stages of development (FM, M, and YT). Significant differences at *p* < 0.05 are represented on bars with different letters.

Fully mature seed extracts in 70% methanol, as well as those from the mature and youngest stages, were evaluated for their NO inhibitory effect in LPS-activated RAW 264.7 cells. Results showed that extracts from YT seeds were far better at lowering NO production than M and FM samples (Figure 6). YT seeds are 0.041 mM lower in nitrite production than LPS and are 0.024 mM lower than mature seed extracts. Previous HPLC results conducted in this study showed that isoquercetin content is higher in YT seeds. Based on the results, it can only be speculated that as the trend of NO production increases with mature seeds (CON < YT < M < FM < LPS), the ability of okra seeds to counteract NO production is optimal at earlier stages of development and slowly decreases as seeds develop (Table S1). Regulation of NO production in plants is essential to the cellular stability and function of cellular components, as unregulated NO production can eventually lead to DNA or protein damage, cell injury, and even apoptosis [31,32,33].

**Figure 7:**
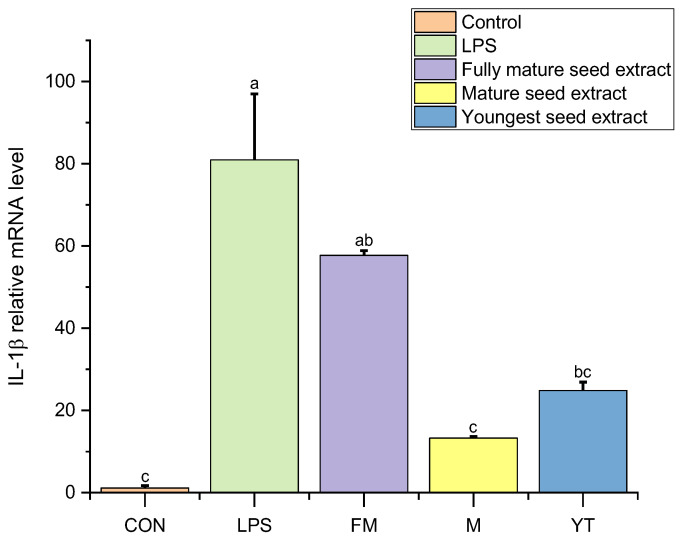
Relative mRNA expression of IL-1β in LPS-induced RAW 264.7 cells treated with FM, M, and YT okra seed extracts. Significant differences at *p* < 0.05 are represented by bars with different letters.

The relative mRNA level of IL-1β was also evaluated (Figure 7). IL-1β mRNA expression was significantly upregulated in LPS-induced RAW 264.7 cells (80.0 ± 5.0-fold, *p* < 0.001 compared to control). Treatment with okra seed extracts significantly reduced LPS-induced IL-1β expression (one-way ANOVA, F = 98.45, df = 4, 10, *p* < 0.001). Post hoc analysis revealed that both mature (12.5 ± 1.5-fold) and fully mature (10.0 ± 1.0-fold) seed extracts exhibited the strongest inhibitory effects, reducing IL-1β expression by approximately 84–88% compared to LPS alone. These two groups were not significantly different from each other (*p* = 0.482). The youngest seed extract showed moderate inhibition (45.0 ± 3.0-fold, 44% reduction), which was significantly less effective than both mature and fully mature extracts (*p* < 0.001) (Table S2).

**Figure 8:**
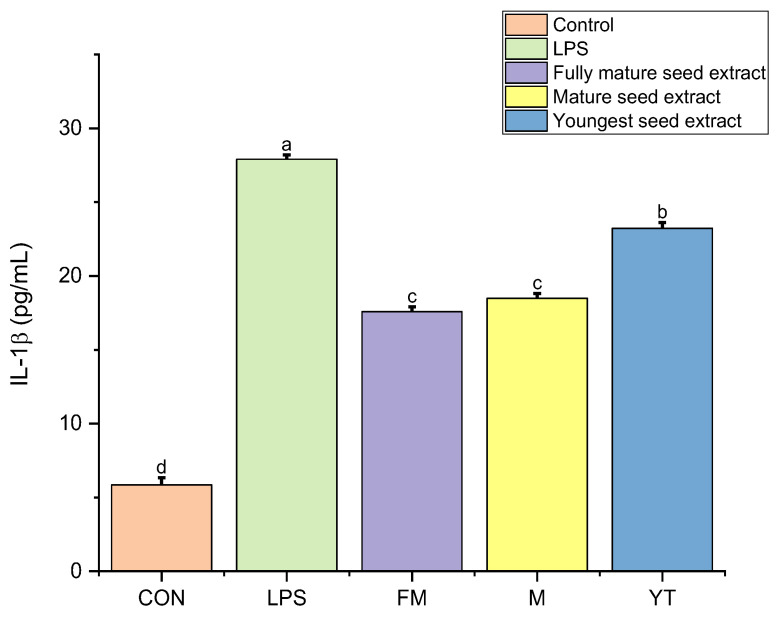
ELISA analysis of IL-1β (B) in LPS-induced RAW 264.7 cells treated with FM, M, and YT okra seed extracts. Significant differences at *p* < 0.05 are represented by bars with different letters.

ELISA analysis was also conducted to confirm the protein level of the results found in the mRNA level (Figure 8). ELISA analysis revealed that LPS stimulation significantly increased IL-1β production to 12.5 ± 0.5 pg/mL compared to the control (6.2 ± 0.1 pg/mL, *p* < 0.001). Treatment with fully mature seed extracts inhibited LPS-induced IL-1β production by 10.32 pg/mL (82.6% reduction), reducing levels to 2.2 ± 0.3 pg/mL. The mature seed extract inhibited IL-1β by 9.42 pg/mL (75.4% reduction), reducing levels to 3.1 ± 0.4 pg/mL. Both fully mature and mature extracts reduced IL-1β concentrations below baseline control levels and were not significantly different from each other (*p* = 0.082). The youngest seed extract showed moderate inhibition (4.5 pg/mL, 36% reduction), reducing IL-1β to 8.0 ± 0.5 pg/mL, which was significantly higher than both mature and fully mature extracts (*p* < 0.001). These findings confirm that mature and fully mature okra seeds possess potent anti-inflammatory activity against the pro-inflammatory cytokine IL-1β (Table S3).

## 3. Discussion

Okra seeds are known to contain mainly quercetin derivatives, catechins and some levels of endogenous gibberellins [7,8]. In this study, various dilutions of methanol were used to extract mature samples of okra seeds. Results showed that a 70% aqueous methanol extract contains the highest levels of total polyphenols and flavonoids compared to other concentrations of 50%, 80%, 90%, and 100% methanol. Hence, based on such results, 70% methanol was used to determine the presence of secondary metabolites in all stages of okra seeds. The efficiency of this solvent is due to the higher solubility of phenols in polar solvents, which results in the higher extraction yield of such compounds [13,34]. Although in other cases acetone was successfully used to extract flavonoids from other plant tissues, in the case of okra seeds, preliminary experiments showed that methanol extraction worked better.

At different stages of development, plants naturally have various levels of bioactive compounds present [28,35,36]; the results in this study regarding the highest TPC and TFC of mature okra seeds show that during such stages, the particular tissues of total polyphenols and total flavonoids have the highest expression and developmental stages can influence the pattern of gene expression related to the biosynthesis of secondary metabolites [28,29].

Regarding the pattern of accumulation between quercetin, isoquercetin, and quercetin-3-O-gentiobiose, it can be observed that isoquercetin accumulates more during the youngest stage of seed development and slowly decreases as the seeds mature. In the case of quercetin-3-O-gentiobiose, it slowly increases after the youngest stage and fully peaks when the seed matures; it decreases after the seeds reach the fully mature stage.

Quercetin, on the other hand, slowly decreases from its already meager content during the youngest stage and starts to slowly increase during the mature stage and increase largely during the fully mature stage. As already mentioned, and as expected, this phenomenon is normal in plant development stages, as the synthesis and accumulation of secondary metabolites were closely associated with differences in stages of development and hence, the content and composition of secondary metabolites also varied [37,38]. A previous study on *Lonicera japonica* had the same observation, where accumulation of chlorogenic acid and luteolin varied at different stages of growth [28,29,30,37,38,39].

The NO assay was also conducted in this study to measure the level of nitrite production. Levels of NO may imply the degree of inflammation and thus are a good indicator for assessing inflammatory processes [40]. LPS-induced macrophages, such as RAW 264.7 cells, in vivo produced varied levels of NO, as presence of bioactive compounds may differ from different stages of okra seed development. LPS is known as a potent activator of macrophages or monocytes and is usually used in inflammation-related studies due to the fact that it generates abundant inflammatory effects [41,42]. It is the major component of cell walls of Gram-negative bacteria, which can also initiate inflammatory processes and stimulate non-immune cells [43]. NO production in this study has been shown to increase as seeds grow to maturity (CON < YT < M < FM < LPS). Such a trend means that there is an increase in NO production as okra seeds develop from the youngest to the fully mature seeds. This efficiency of okra seeds to counteract NO production at earlier stages can be related to the bioactive compound isoquercetin, as the latter is most prevalent in the youngest seeds and eventually isoquercetin decreases as seeds fully mature in 70% methanol extract (YT > Y > I > M > FM).

IL-1β is a pro-inflammatory cytokine which is known to promote activation of T cells, maturation of B cells, and activity of natural killer cells. It is also involved in several neurophysiological and neuroimmunological activities in the central nervous system, the production and activity of which involve multiple regulatory steps including transcription, translation, cellular cleavage and secretion [44,45]. It has been found in the post-mortem striata and cerebrospinal fluid of patients with Parkinson’s disease and is closely associated with the activation of astrocytes leading to reactive oxygen and nitrogen species production [41,45,46,47]. Overexpression of IL-1β may lead to autoimmune abnormality, fever, or pain [3,48].

Beyond quercetin-3-O-gentiobiose, quercetin itself, which accumulated to its highest level in fully mature seeds, has been independently associated with a range of protective bioactivities relevant to metabolic and inflammatory disease. Related anthocyanins have been shown to ameliorate hyperglycemia and improve insulin sensitivity in diabetic models [49], and dietary flavonoids more broadly exhibit antidiabetic actions [50]. Anthocyanins have been reported to regulate adipocyte function relative to the prevention of metabolic syndrome [51]. Quercetin specifically has demonstrated a hepatoprotective effect against endoplasmic reticulum stress and inflammation [52], has been shown to alleviate oxidative damage and inflammatory stress via modulation of the p38 MAPK pathway [53], and has been reported to protect against nickel-induced DNA methylation and inflammation through the Nrf2/HO-1 and p38/STAT1/NF-κB pathways [54]. These findings lend support to the potential health relevance of elevated quercetin content observed in mature and fully mature okra seeds in this study.

Moreover, the addition of okra seed extracts generally lowered the relative expression of IL-1β. This is pharmacologically significant as many diseases are closely related to inflammatory processes. The highest inhibition can be found in extracts of M seed samples, which are 67.68 points lower than the LPS-induced samples. It can be noted that in M seeds, the major flavonoid found via HPLC evaluation is that of quercetin-3-O-gentiobiose. Quercetin-3-O-gentiobiose in previous studies was known to possess antioxidative properties and has also been known to alleviate vascular impairments and attenuate levels of other inflammatory cytokines, such as interleukin-6 (IL-6), tumor necrosis factor-alpha (TNF-α), and monocyte chemoattractant protein-1 (MCP-1). It was hence known to contain anti-fatigue and vasoprotective effects [25]. Such potent anti-inflammatory ability of quercetin-3-O-gentiobiose is worth accounting for in this part of the study.

## 4. Materials and Methods

### 4.1. Preparation of Okra Seed Samples

Okra seeds were procured from East-West Seeds, Philippines. Each seed was planted in a pot with loam soil and was placed in a chamber under 150 µmol m^−2^ s^−1^ PPFD white light in a 16/8 h photoperiod, 75% humidity and a temperature of 25 °C. Okra fruits were then harvested from the day they began to emerge or the day after pollination and were recorded as follows: youngest (YT), 5 days old; young (Y), 7 days old; intermediate (I), 9 days old; mature (M), 11 days old; and fully mature (FM), 20 days old (Figure 1). Seeds from fruits at different levels of development were then taken out from the pods and stored at −60 °C.

### 4.2. Preparation of Okra Seed Extract

Okra seed extracts from different levels of fruit development were prepared by freeze-drying 300 mg of samples using liquid nitrogen. The resulting powder was added to 3 mL of 70% methanol. Samples were sonicated in an ultrasonic bath (Danbury, CT, USA) for 5 min and were left in an orbital shaker (LaboGene, Seoul, Republic of Korea) for 15 min at a speed of 20 rpm. Samples were then centrifuged for 10 min at 10,000 rpm. The supernatant was filtered through a sterile syringe (Sercrim, Seoul, Republic of Korea) and a 0.45 µm Whatman syringe filter. Filtered extracts were then stored at −60 °C until use. For further HPLC analysis, the stored extract was placed in a 10 mL test tube and was subjected to freeze-drying (Operon Co., Ltd., Seoul, Republic of Korea) for 48 h. Dried samples were weighed and diluted with 70% methanol and were again filtered as described above.

### 4.3. Determination of Total Polyphenol Content in Okra Seeds

Total polyphenol content (TPC) was measured as described previously by [55] with some modifications. Gallic acid was used as a standard and was prepared by diluting 1 mg of gallic acid powder in 1 mL distilled water (DW). In a 1.5 mL Eppendorf tube, either 10 µL of okra seed extract from 70% methanol or a gallic acid standard solution of various concentrations was separately added to 250 µL of DW together with 160 µL of a 10% Folin–Ciocalteu reagent. This mixture was left for 5 min at room temperature (RT) and 300 µL of 10% sodium carbonate solution was added to the tube. This was left at RT for 30 min. Sample absorbance was then measured in a spectrophotometer (BioTek Instruments, Inc., Winooski, VT, USA) at 750 nm. The resulting TPC was expressed as mg of gallic acid equivalent (GAE/g) of the sample extract.

### 4.4. Determination of Total Flavonoid Content in Okra Seeds

Total flavonoid content (TFC) was measured as described previously by [55] with some modifications using the aluminum chloride colorometric method and isoquercetin as the standard. Isoquercetin was prepared by diluting 1 mg of powder in 1 mL DW. In a 1.5 mL Eppendorf tube, 25 µL of the okra seed extract from either 70% methanol or solely the isoquercetin standard solution with different concentrations was separately added with 400 µL DW and 30 µL of 5% sodium nitrite. After leaving it for 5 min, 30 µL of 10% AlCl3 was added, and the mixture was incubated at RT for 6 min. Another 240 mL DW was then added together with 200 µL of 1 molar (M) sodium hydroxide. The absorbance of the solution was measured at 510 nm. The resulting TFC was expressed as mg of isoquercetin equivalence (ISE)/g of the sample extract.

### 4.5. DPPH Free Radical Scavenging Activity of Okra Seeds

DPPH free radical scavenging activity was conducted as previously described with some modifications [56]. The DPPH working solution in 0.1 mM was diluted in ethanol. DPPH solution in 150 µL was separately added to 5 µL of okra seed extracts from 70% methanol or ethanol alone as the control solution. This mixture was then placed in the dark for 30 min and absorbance was measured in a spectrophotometer at 515 nm. Percentage of DPPH scavenging activity was calculated as follows.: DPPH radical scavenging activity (%) = [(Ac − As)/ Ac] × 100. Here, Ac and Ab are the absorbance of the control and sample, respectively.

### 4.6. ABTS+ Radical Cation Scavenging Activity of Barley Sprouts and Okra Seeds

ABTS+ radical cation scavenging activity was conducted as described previously with some modifications [56]. ABTS stock solution was prepared with 5 mL of 7 mM ABTS and 88 µL of 140 mM potassium persulfate. The stock solution was kept in the dark for 16 h at RT, 1 mL of which was diluted with 88 mL ethanol. In an Eppendorf tube, 3 µL of the okra seed sample extract from 70% methanol was then added to a 1 mL ABTS solution for 3 min. Sample absorbance was measured at 734 nm. Percentage of ABTS+ radical scavenging activity was calculated as follows: ABTS radical scavenging activity (%) = [(Ac − As)/Ac] × 100. Here, Ac and Ab are the absorbance of the control and sample, respectively.

### 4.7. Quantification of Quercetin and Isoquercetin Content in Okra Seeds

HPLC analysis was performed as described previously by [57] with some modifications. The Agilent 1260 VWD HPLC system (Agilent, Santa Clara, CA, USA) was used with a Capcell Pak C18 UG120 (4.6 × 250 mm, 5 μm) column maintained at 40 °C. The mobile phase was composed of 0.1% formic acid in DW as solvent A and 100% methanol as solvent B. The binary gradient elution was as follows: 0–18 min, 0–60% B; 19–20 min, 60–100% B; 21–22 min, 60% B; and 23–29 min, 20% B. The flow rate was 0.8 mL/min with a 10 µL injection volume and absorbance was measured at 360 nm. Each sample extract was tested in triplicate. Each standard stock solution of 200 mg/kg (quercetin-3-4′-di-glucoside, isoquercetin, quercetin-4′-glucoside, quercetin, kaempferol) was diluted with 70% methanol to 0.5, 2, 5, 10, 20, and 100 mg/kg, and used as a standard solution for the preparation of the calibration curve. Quercetin and isoquercetin content were determined by comparing retention time to that of the set standards.

### 4.8. Quantification of Quercetin-3-O-Gentiobiose Content in Okra Seeds

HPLC analysis was performed using the Agilent 1100 series HPLC system (Agilent, CA, USA) with a YMCA-Triart C18 (250 × 4.6 mm; i.d., 5 µm) column maintained at 25 °C. Solvent A for the mobile phases is acetonitrile, while solvent B is DW with 0.1% acetic acid. The gradient elution was as follows: 0–10 min, 0–90% B; 11–20 min, 80% B; 21–30 min, 70% B; 31–40 min, 60% B; 41–50 min, 10% B; and 51–65 min, 90% B. The absorbance was measured at 354 nm at a flow rate of 1 mL/min and a 5 µL injection volume. Quantification of quercetin-3-O-gentiobiose was determined by comparing the retention time to that of the set standards and calculating the area under the identified standard curve. Samples were taken from 70% methanol extraction.

### 4.9. Cell Culture of RAW 264.7

The RAW 264.7 cell line obtained from ATCC (Manassas, VA, US) was cultured in Dulbecco’s modified Eagle’s medium (DMEM) supplemented with 10% fetal bovine serum (FBS), 100 µg/mL streptomycin, 100 U/mL penicillin, and 1 M 4-(2-hydroxyethyl)-1-piperazineethanesulfonic acid (HEPES). At 37 °C, cell cultures were incubated in a 5% carbon dioxide humidified incubator. After seeding the cells in a 6-well plate, cells were treated separately with 50 µg/mL of okra samples from 70% methanol extracts from FM, M, and YT seeds for a 24 h incubation. This was followed by another 24 h incubation with the addition of 20 ng/mL of LPS in phosphate-buffered saline (PBS). The culture supernatant was isolated to measure levels of nitric oxide (NO), relative mRNA levels, and protein levels of IL-1β. The culture medium was changed three times a week.

### 4.10. Measurement of NO and Pro-Inflammatory Cytokine IL-1β in Okra Seed Extracts

A cell culture supernatant of 100 µL was mixed with 100 µL of Griess reagent made from 2.5% phosphoric acid containing 0.1% N-(1-naphthyl) ethylenediamine dihydrochloride and 1% sulfanilamide. The mixture was incubated at 37 °C for 10 min and wavelength detection was assessed at 540 nm. The levels of nitrite synthesis were determined using a standard curve. Sample extracts were taken from 70% methanol extraction.

The cell culture supernatant was used for the separate detection of pro-inflammatory cytokine IL-1β through the enzyme-linked immunosorbent assay (ELISA) following the manufacturer’s kit protocol (R&D Systems, Rockford, IL, USA). Levels of detection were quantified using a standard curve as provided with the kit. Experiments were performed in triplicate. Sample extracts were taken from 70% methanol extraction.

### 4.11. RNA Isolation and Quantitative Real-Time PCR (qRT-PCR) of IL-1β in Okra Seed Extracts

Total RNA was isolated from the cultured RAW 264.7 cell line using the TRIzol reagent (Life Technologies, Rockville, MD, USA). To each well of cells, TRIzol was added, followed by chloroform after 5 min. The supernatant was then mixed with an equivalent volume of isopropanol. The extracted mRNA was diluted to 400 ng/mL in nuclease-free water. cDNA was synthesized by reverse transcription using a high-capacity RNA-to-cDNA synthesis kit (Hoffmann La Roche, Basel, Switzerland). Quantification of RNA was conducted using the Step-One-Plus RT-PCR system (Hoffmann La Roche, Basel, Switzerland) along with the Universal Probe Library (UPL) probe method. Amplifications were performed at 95 °C with a 10 min template denaturation, followed by 40 cycles at 95 °C for 10 s each and 60 °C for 30 s each. The comparative CT method was used for the quantification of relative mRNA expression levels normalized against glyceraldehyde 3-phosphate dehydrogenase (GAPDH). The forward and reverse primers used are shown in Table 5.

### 4.12. Statistical Analysis

Statistical analyses were performed using OriginPro 2022 (OriginLab Corporation, Northampton, MA, USA). All experiments were conducted in triplicate independent biological replicates (*n* = 3), and results were expressed as the mean ± standard error of the mean (SEM) unless otherwise specified. Normality across the different developmental stages was assessed using the Shapiro–Wilk test, while Levene’s test was employed to evaluate the homogeneity of variances. One-way ANOVA was applied to samples that exhibited both normal distribution and equal variances across the different developmental stages of okra. In cases where normality was satisfied but variances were unequal, Welch’s ANOVA was used instead.

Moreover, the Kruskal–Wallis test was employed for samples that violated the assumptions of normality and homogeneity of variances. A significance level of α = 0.05 was set for all statistical tests. Consequently, *p*-values < 0.05 were deemed statistically significant, except for the one-way ANOVA results, for which a more stringent threshold of *p* < 0.01 was applied.

## 5. Conclusions

In conclusion, okra seed samples from mature seeds were found to contain the highest TPC, TFC, and antioxidant activities using 70% methanol as the solvent. Mature seeds were found to contain a high quantity of quercetin, while the youngest and fully mature seeds contained a high quantity of isoquercetin and quercetin-3-O-gentiobiose, respectively. Mature seed samples were also found to have the highest anti-inflammatory effects against pro-inflammatory cytokine IL-1β in LPS-induced RAW 264.7 cells. This result further confirms the anti-inflammatory effects of quercetin-3-O-gentiobiose, which is the major flavonoid present in mature okra seed extracts. Hence, the determination of secondary metabolites in okra seeds from various stages of development is deemed an essential study due to its promising pharmacological and biological significance. Further studies are highly recommended to enhance the efficiency of secondary metabolite extraction in okra seeds.

## Figures and Tables

**Table 1:** Total polyphenol and flavonoid content of mature okra seeds extracted in various methanol concentrations.

Solvent	TPC (mg GAE/g)	TFC (mg ISE/g)
100% Methanol	293.30 ± 3.71 ^g^	78.04 ± 1.44 ^g^
90% Methanol	567.79 ± 5.06 ^e^	121.83 ± 2.59 ^d^
80% Methanol	684.18 ± 7.82 ^bc^	136.19± 1.44 ^c^
70% Methanol	678.34 ± 6.15 ^c^	152.70 ± 1.24 ^b^
50% Methanol	510.64 ± 2.21 ^f^	97.42 ± 1.90 ^e^

Values are expressed as mean ± SEM (*n* = 3). Values within the same column with different superscript letters are significantly different at *p* < 0.05.

**Table 2:** Quercetin content across okra seed developmental stages with statistical comparison.

Stage	Replicates (*n*)	Mean ± SEM (mg/kg)	Shapiro–Wilk Test	Normality	Welch’s ANOVA
Fully Mature	3	148.14 ± 6.20 ^a^	0.353	Yes	*F* = 156.23, *p* < 0.001; *Significant*
Mature	3	29.02 ± 0.46 ^c^	0.735	Yes	
Intermediate	3	22.86 ± 0.65 ^d^	0.930	Yes	
Young	3	30.63 ± 1.31 ^c^	0.504	Yes	
Youngest	3	42.05 ± 0.77 ^b^	0.726	Yes	

Different superscript letters indicate significant differences using the Games–Howell post hoc test (*p* < 0.05).

**Table 3:** Isoquercetin content across okra seed developmental stages with statistical comparison.

Stage	Replicates (*n*)	Mean ± SEM (mg/kg)	Shapiro–Wilk Test	Normality	Kruskal–Wallis
Fully Mature	3	34.64 ± 2.29 ^b^	0.826	Yes	*H* = 12.54, *p* = 0.014; *Significant*
Mature	3	3953.37 ± 308.26 ^ab^	0.325	Yes	
Intermediate	3	3956.93 ± 59.36 ^ab^	0.747	Yes	
Young	3	4976.11 ± 181.85 ^a^	0.005	No	
Youngest	3	5205.02 ± 48.54 ^a^	0.930	Yes	

Different superscript letters indicate significant differences using Dunn’s post hoc test with Bonferroni correction (*p* < 0.05).

**Table 4:** Quercetin-3-*O*-gentiobiose content across okra seed developmental stages with statistical comparison.

Stage	Replicates (*n*)	Mean ± SEM (mg/kg)	Shapiro–Wilk Test	Normality	One-Way ANOVA
Fully Mature	3	15.30 ± 14.80 ^a^	0.504	Yes	*F* = 98.45, *p* < 0.001; *df* = 4, 10; *Significant*
Mature	3	12,396.48 ± 77.54 ^e^	0.920	Yes	
Intermediate	3	11,086.93 ± 49.54 ^d^	0.970	Yes	
Young	3	9538.85 ± 57.47 ^c^	0.910	Yes	
Youngest	3	2768.06 ± 10.31 ^b^	0.960	Yes	

Different superscript letters indicate significant differences using Tukey’s HSD post hoc test (*p* < 0.001 for all comparisons).

**Table 5:** Primer sequences for qRT-PCR.

Cell Line	Gene Name	Oligonucleotide Sequence	Amplicon Size (bp)	Efficiency (%)
RAW 264.7	*GAPDH*	F: 5′-AAGAGGGATGCTGCCCTTAC-3′ R: 5′-CCATTTGTCTACGGGACGA-3′	112	98.2
	*IL-1β*	F: 5′-AGTTGACGGACCCCCAAAAG-3′ R: 5′-AGCTGGATGCTCTCATCAGG-3′	123	101.5

## Data Availability

The original contributions presented in this study are included in the article/Supplementary Material. Further inquiries can be directed to the corresponding author.

## Supplementary Materials

The following supporting information can be downloaded at https://www.mdpi.com/article/10.3390/ijms27157002/s1.
