# Supplementary material for: Evaluation of Secondary Metabolites in Various Stages of Okra Seed Development and Their Anti-Inflammatory Potential Against IL-1ß
Source: Int J Mol Sci. 2026 Aug 4;27(15):7002. doi: 10.3390/ijms27157002 (PMC13466827; doi:10.3390/ijms27157002)
Supplement: Supplementary file 1 [file ijms-27-07002-s001.zip › ijms-4116151-supplementary.pdf]

Evaluation of Secondary Metabolites in Various stages of Okra Seed Development and its Anti-inflammatory Potential against IL-1ß.

Lorrenne Caburatan [1], and Joonho Park [2,3] *

[1]Department of Biological Sciences, Mindanao State University - Iligan Institute of Technology, 9200 Iligan City, Philippines

[2]Department of Fine Chemistry, Seoul National University of Science and Technology, 232-Gongneung-ro, Nowon-gu, 01811 Seoul, Republic of Korea

[3]Center for Functional Biomaterials, Seoul National University of Science and Technology, 232-Gongneung-ro, Nowon-gu, 01811 Seoul, Republic of Korea

* **Correspondence:** jhpark21@seoultech.ac.kr

List of Figures

Figure S1. HPLC chromatograms of quantified quercetin and isoquercetin from okra seeds in various stages of development using 70% methanol (D-FM; E-M; F-I; G-Y; H-YT) with the mix standard of highest concentration (A) and the standard quantitative curve (B-quercetin; C-isoquercetin).

Figure S2. HPLC chromatograms of quantified quercetin-3-*O*-gentiobiose from okra seeds in various stages of development using 70% methanol (C-FM; D-M; E-I; F-Y; G-YT) with the standard of highest concentration (A) and the standard quantitative curve (B).

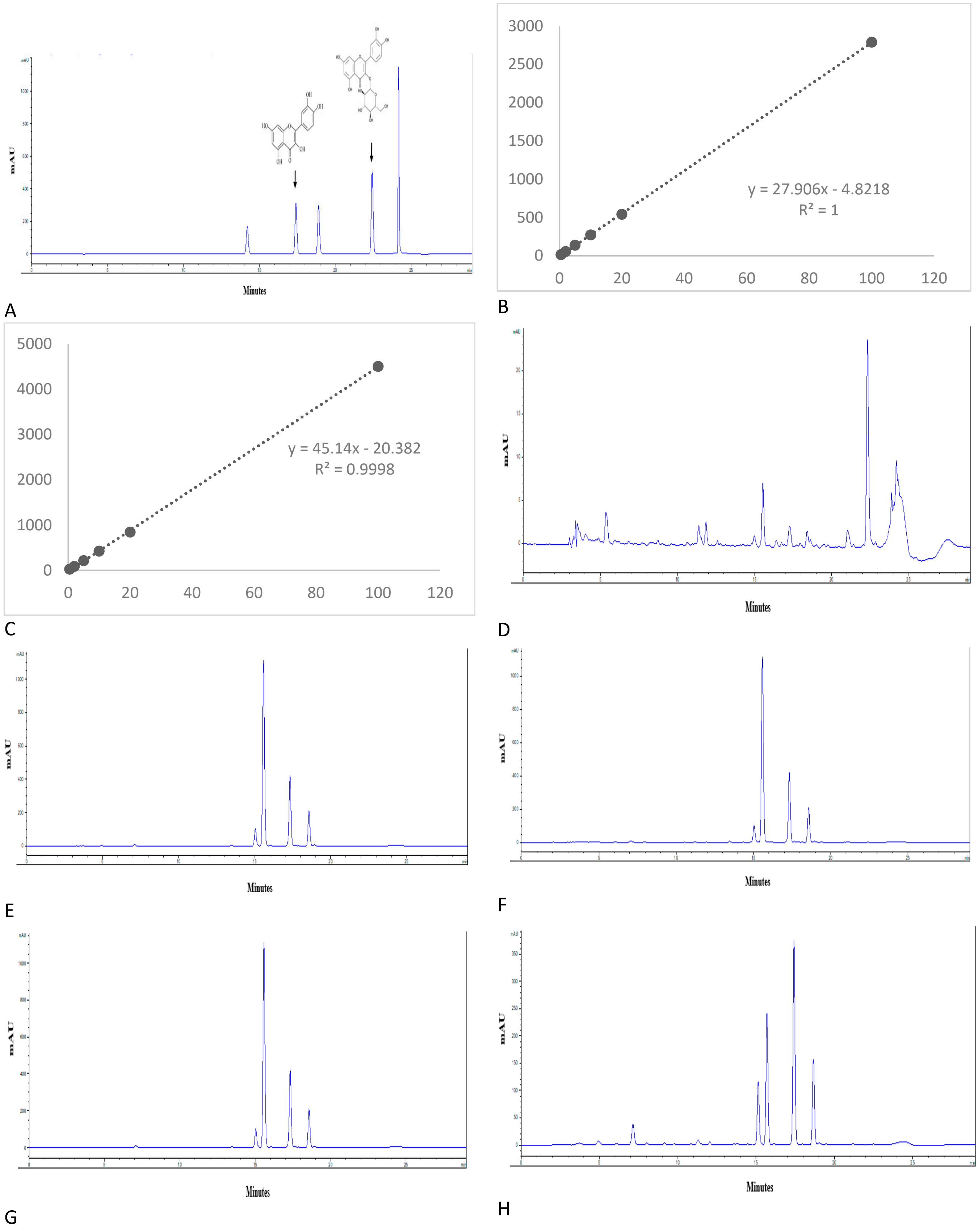

Figure S1. HPLC chromatograms of quantified quercetin and isoquercetin from okra seeds in various stages of development using 70% methanol (D-FM; E-M; F-I; G-Y; H-YT) with the mix standard of highest concentration (A) and the standard quantitative curve (B-quercetin; C-isoquercetin).

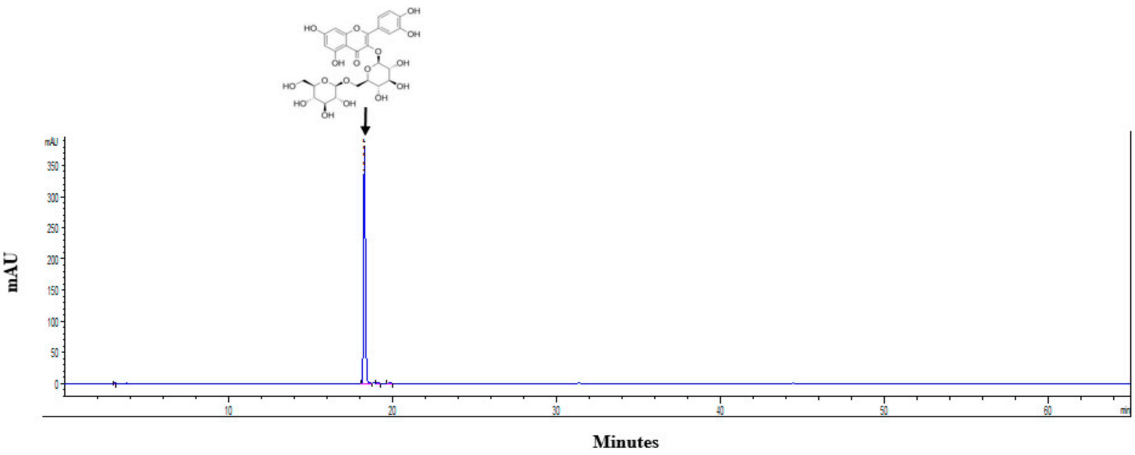

A

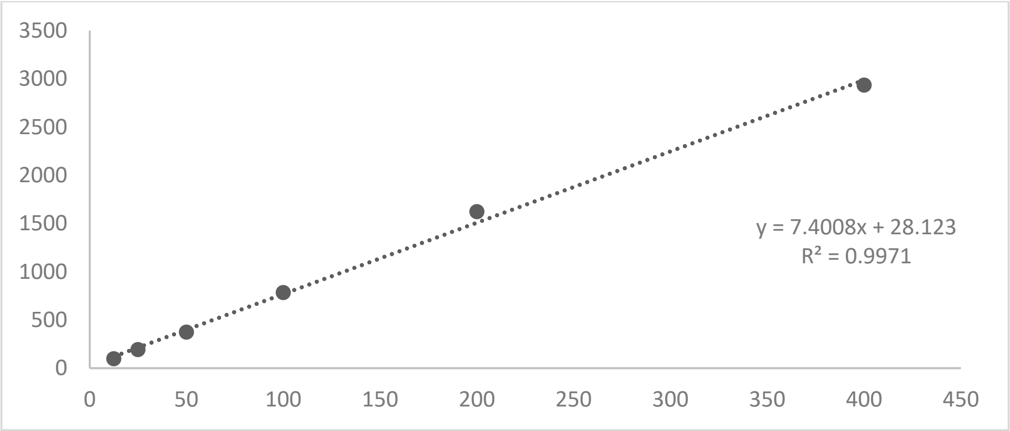

B

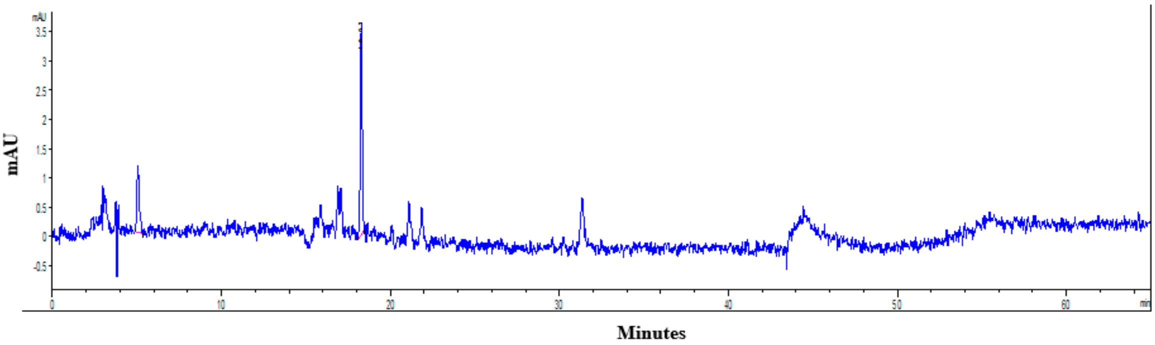

C

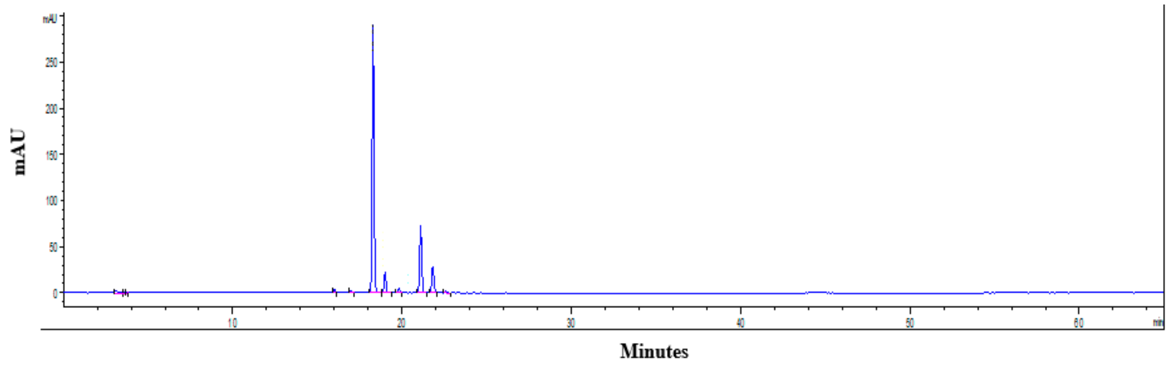

D

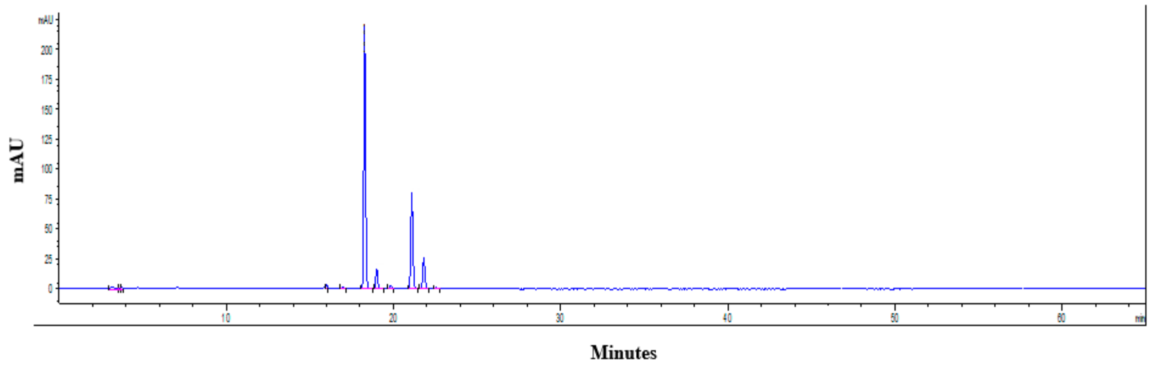

E

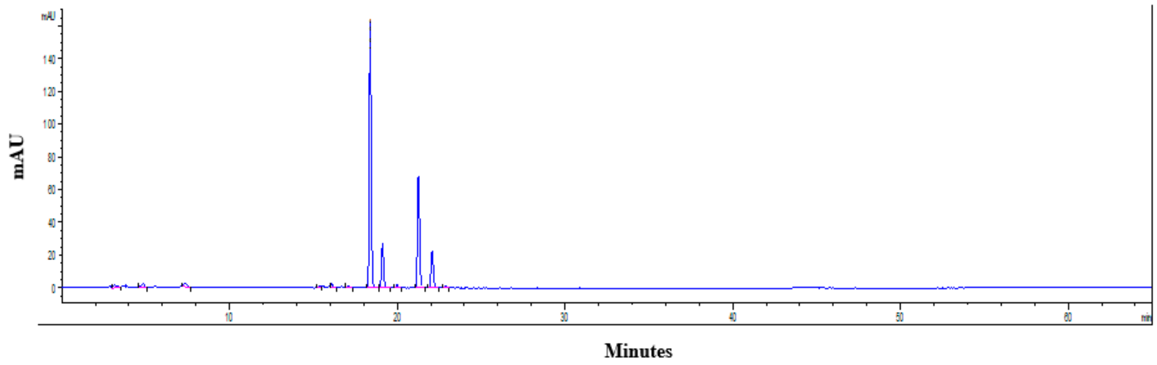

F

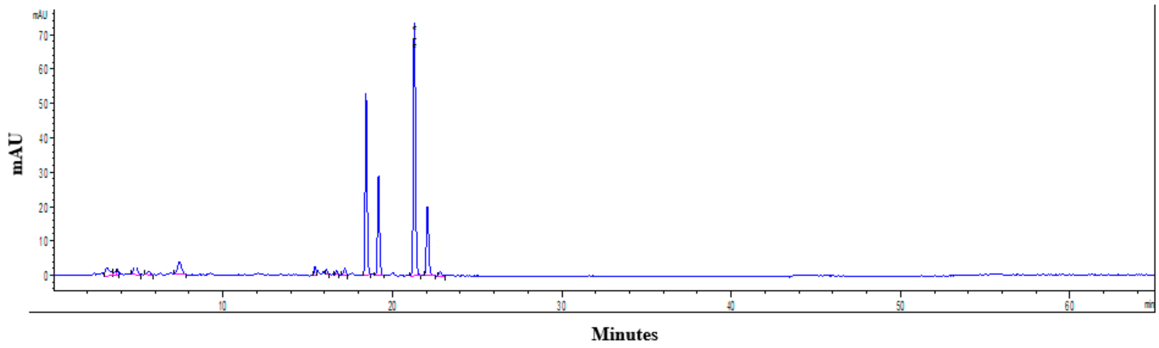

G

Figure S2. HPLC chromatograms of quantified quercetin-3-*O*-gentiobiose from okra seeds in various stages of development using 70% methanol (C-FM; D-M; E-I; F-Y; G-YT) with the standard of highest concentration (A) and the standard quantitative curve (B).

List of Tables

Table S1. Nitric oxide levels in LPS-induced RAW 264.7 cells consequently added with okra seed extracts in 70% methanol from different stages of development (FM, M, and YT).

Table S2. Relative mRNA expression of IL-1β in LPS-induced RAW 264.7 cells treated with FM, M, and YT okra seed extracts.

Table S3. ELISA analysis of IL-1β (B) in LPs-induced RAW 264.7 cells treated with FM, M, and YT okra seed extracts.

Table S1. Nitric oxide levels in LPS-induced RAW 264.7 cells consequently added with okra seed extracts in 70% methanol from different stages of development (FM, M, and YT).

Treatment	Replicates (n)	Mean ± SD (μM)	Shapiro-Wilt Test	Normality	ANOVA
Control	3	2.50 ± 0.10^{a}	1.000	+	F = 98.45, *p < 0.001;*
LPS	3	12.50 ± 0.50^{b}	0.920	+	*df = 4, 10*
LPS + Fully Mature	3	10.00 ± 0.50^{c}	0.910	+	*Significant*
LPS + Mature	3	9.50 ± 0.50^{c}	0.970	+	
LPS + Youngest	3	5.00 ± 0.20^{d}	0.960	+	

** Different superscript letters (a, b, c, d) indicate significant differences by Tukey HSD post-hoc test (p < 0.05).*

Table S2. Relative mRNA expression of IL-1β in LPS-induced RAW 264.7 cells treated with FM, M, and YT okra seed extracts.

Treatment	Replicates (n)	Relative IL-1β mRNA Expression (fold change)	SD	Inhibition relative to LPS (%)
Control	3	1.00	0.10	---
LPS	3	80.00	5.00	---
LPS + Fully Mature	3	10.00	1.00	87.50
LPS + Mature	3	12.50	1.50	84.40
LPS + Youngest	3	45.00	3.00	43.80

Table S3. ELISA analysis of IL-1β (B) in LPs-induced RAW 264.7 cells treated with FM, M, and YT okra seed extracts.

Treatment	Replicates (n)	Mean ± SD (pg/mL)	Shapiro-Wilt Test	Normality	ANOVA
Control	3	6.20 ± 0.10^{a}	---	Constant	F = 98.45
LPS	3	12.50 ± 0.50^{b}	0.920	+	*p < 0.001;*
LPS + Fully Mature	3	2.20 ± 0.30^{c}	0.537	+	*df = 4, 10*
LPS + Mature	3	3.10 ± 0.40^{c}	0.743	+	*Significant*
LPS + Youngest	3	8.00 ± 0.50^{d}	0.910	+	

** Different superscript letters (a, b, c, d) indicate significant differences by Tukey HSD post-hoc test (p < 0.05).*
